# Enhancing surgical care for torture survivors: healing trauma, promoting recovery, and advancing best practices

**DOI:** 10.1186/s12893-025-03042-0

**Published:** 2025-08-01

**Authors:** Ana Carla S. P. Schippert, Ellen Karine Grov, Juha Silvola, Ann Kristin Bjørnnes

**Affiliations:** 1 Institute of Nursing and Health Promotion, Oslo Metropolitan University, Oslo, Norway; 2 Akershus University Hospital, Lørenskog, Norway; 3 Institute of Clinical Medicine, Campus Ahus, University of Oslo, Campus Ahus, Oslo, Norway

**Keywords:** Torture survivors, Surgery, Knowledge, Awareness, Trauma-informed care

## Abstract

**Background:**

A significant proportion of refugees and asylum seekers have experienced torture, leading to complex, long-term physical and psychological health needs. Many require surgical care for trauma-related injuries but often face barriers such as fear, mistrust, and a lack of trauma-specific knowledge among healthcare professionals. In surgical settings, the absence of trauma-informed practices can contribute to retraumatisation, misdiagnosis, and inadequate care. This narrative review provides an overview offering clinical insights to underscore the importance of trauma-informed, interdisciplinary approaches in the surgical care of torture survivors.

**Literature search:**

This narrative review is based on a targeted literature search conducted across PubMed, Google Scholar, Scopus, and Web of Science between August 2023 and January 2024. Relevant articles were identified through keyword-based searches and manual reference screening. The review focused on literature addressing somatic and surgical care for torture survivors, with an emphasis on trauma-informed approaches. An iterative process was used until thematic saturation was achieved.

**Review of literature:**

Torture survivors frequently suffer from chronic physical conditions—such as brain injuries, fractures, contractures, hearing loss, and genitourinary damage—that may require surgical intervention. Despite these needs, they often face significant barriers to care, including fear, mistrust, and a lack of trauma-specific competence among healthcare professionals. The literature highlights that many survivors receive conflicting diagnoses and inadequate treatment within surgical services, contributing to confusion and loss of trust. Surgical teams are often unprepared to identify signs of torture or manage their complex psychological and physical effects. A trauma-informed, interdisciplinary approach is essential to address these gaps.

**Conclusions:**

Surgical care for torture survivors must integrate trauma-informed practices at all stages to ensure safety, dignity, and effective treatment. Interdisciplinary collaboration is essential. Further research is needed to evaluate these approaches and understand their long-term impact on surgical treatment outcomes.

## Introduction

War trauma and torture are tremendously distressing experiences, with torture victims ranking among the most susceptible categories of refugees [1]. Approximately 35% of refugees and asylum seekers have been tortured [2, 3], but this number is increasing [4]. The United Nations (UN) defines torture as [5].

Any act that deliberately inflicts severe pain or suffering, whether physical or mental, on an individual for purposes such as eliciting information or a confession from that individual or a third party, punishing them for an act they or a third party have committed or are suspected of committing, intimidating or coercing them or a third party, or for any reason based on discrimination of any kind.

Further, ongoing conflicts—such as wars, civil unrest, and political instability—significantly contribute to the rise in the number of torture survivors [4, 6]. These situations often create environments where human rights are violated, and individuals are subjected to torture as a means of control, intimidation, or punishment [6]. Additionally, the breakdown of law and order during conflicts leads to the proliferation of violent practices targeting vulnerable groups and individuals. This perpetuates cycles of trauma, displacement, and suffering, thus leaving survivors with long-lasting physical, emotional, and psychological scars [7, 8]. 

Torture survivors have a heightened propensity for suffering from chronic mental and physical health disorders, including post-traumatic stress disorder (PTSD) [9, 10]. Many survivors refrain from disclosing their torture experiences to healthcare professionals because of fear, embarrassment, shame, inadequate support, and mistrust [11–15]. Particularly in somatic fields, such as surgical units, healthcare professionals possess insufficient understanding and training to identify torture survivors within the healthcare system [16–18]. Recognising torture survivors is the initial step in delivering appropriate healthcare to this patient group. The identification of torture survivors in the surgical clinical context appears to rely on self-identification, although members of the surgical team often lack the requisite expertise for this process [17–20]. 

Torture survivors have intricate health requirements stemming from a blend of physical and psychological symptoms, alongside comorbidity and somatisation, thus necessitating specialised medical proficiency [21, 22]. Chronic physical ailments stemming from torture—including cerebral injuries, fractures, contractures, hearing deficits, and issues related to urine and defecation due to beatings, imposed positions, electric shocks, burns, sexual violence, and other methods—may necessitate surgical treatment [23–25]. 

In addition to providing treatment, surgeons and other members of the surgical team have an ethical and professional responsibility to recognise, document, and report signs of torture and other contributing to efforts to prevent and ultimately end such abuses [25]. 

Survivors of torture pursuing surgical treatment report receiving conflicting diagnoses and undergoing multiple ineffective treatments, thus resulting in confusion and disillusionment [17, 21, 26–28]. 

Because of the interplay of numerous physical and psychological factors, as well as the profound psychological consequences of torture, survivors often present a complex clinical picture that can be challenging to interpret, even for the most experienced surgeons [29]. Given this complexity, it is crucial for surgeons and other healthcare professionals to adhere to existing recommendations to improve treatment outcomes and prevent complications, such as the reactivation of earlier trauma during healthcare interventions [29]. Due to the multidimensional nature of the conditions presented by torture survivors, a comprehensive and interdisciplinary approach is essential to effectively diagnose and address each survivor’s specific needs [30]. 

The primary aim of this study is to synthesise existing information and literature on torture survivors [31], highlighting the widespread impact of torture and the crucial role of surgical care, including the frequent need for surgical intervention. It seeks to enhance the awareness and understanding of torture’s complex repercussions among surgeons and surgical teams. By contributing to the existing body of knowledge, the paper advocates for a more informed, compassionate surgical practice and calls for the integration of trauma-informed care (TIC) in surgical departments, where such an approach is currently lacking [32–35]. 

## Methods

The literature cited in this review includes articles already familiar to the author, supplemented by additional relevant papers identified through reference searches. Due to the specific and narrowly defined focus of the review, a formal systematic literature search was not conducted. However, to ensure a comprehensive and relevant selection of sources, four major online databases—PubMed, Google Scholar, Scopus, and Web of Science—were searched from August 2023 to January 2024.

The review aimed to capture a diverse range of studies that addressed key aspects of somatic and surgical care for torture survivors. A targeted search strategy was employed, combining carefully selected keywords such as “torture survivors,” “somatic”, “surgical treatment,” “anesthesia,” “torture methods,” “sequelae,” “pain,” “trauma-informed care,” “retraumatisation,” “communication,” and “best practices.” These terms were selected to reflect the main domains relevant to surgical management in this population. Multiple keyword combinations were applied to maximise the retrieval of relevant literature.

The selection process continued with manual identification and review of additional publications cited in the initially retrieved articles. This iterative approach was repeated until a saturation point was reached; the saturation point is defined as the stage at which no new themes, perspectives, or significant findings relevant to surgical care for torture survivors emerged [36].

## Results

The narrative review identified a range of physical, psychological, and procedural challenges in the surgical care of torture survivors. The literature consistently revealed that survivors often present with complex injuries resulting from diverse methods of torture, including fractures, burns, soft tissue damage, and chronic pain syndromes. These injuries frequently necessitate surgical intervention, often followed by long-term rehabilitative support.

A theme identified across a few of the reviewed studies was the absence of trauma-informed approaches within surgical settings. Survivors described experiences of retraumatisation during preoperative assessments, anaesthesia administration, and postoperative care—particularly when healthcare providers lacked awareness or training in the clinical and psychological impacts of torture.

The key best practices emphasised in the literature included the importance of multidisciplinary care, the integration of psychological support, and the consistent use of trauma-informed communication. However, the review also revealed a notable lack of standardised guidelines tailored to the surgical management of torture survivors.

Overall, the findings highlight an urgent need for greater clinical awareness, targeted training, and institutional preparedness to ensure ethical, compassionate, and effective surgical care for this vulnerable population.

## A case study

From among the findings, a fictitious case was developed to illustrate key concepts, ground the discussion in a practical context, and guide the reader through the article’s core argument.


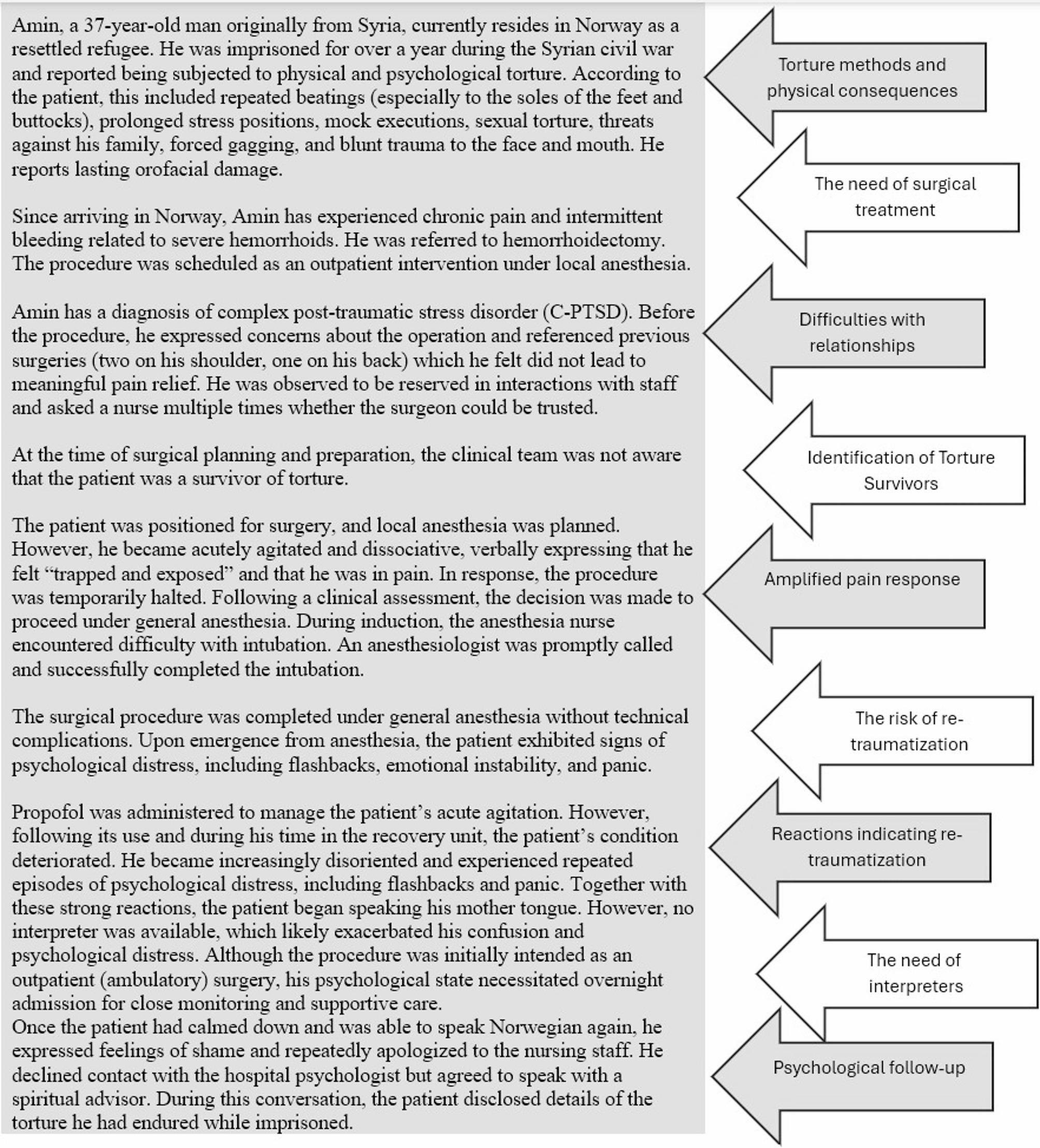


## Addressing the consequences of torture: A comprehensive approach for surgical teams

The methods of torture encompass both physical and psychological dimensions, and it is often challenging to draw a clear boundary between the two. Similarly, the consequences of torture exhibit a complex interplay between physical and psychological effects, thus making it difficult to distinctly separate them [4]. When addressing the aftermath of torture, surgeons and other members of surgical teams primarily focus on treating the physical injuries sustained by survivors. However, it is important to recognise and understand the psychological repercussions that accompany physical trauma. While surgeons and the other members of the team may not directly treat psychological issues, they must operate with an awareness of the patient’s mental state [28]. Comprehending the psychological ramifications of torture is a form of TIC and may assist caregivers in adjusting care, thereby improving patient interactions, enhancing the overall care experience, and facilitating better recovery outcomes [35]. This perspective helps create a supportive environment that acknowledges the full scope of the survivor’s suffering and fosters comprehensive healing.

**Fig. 1:**
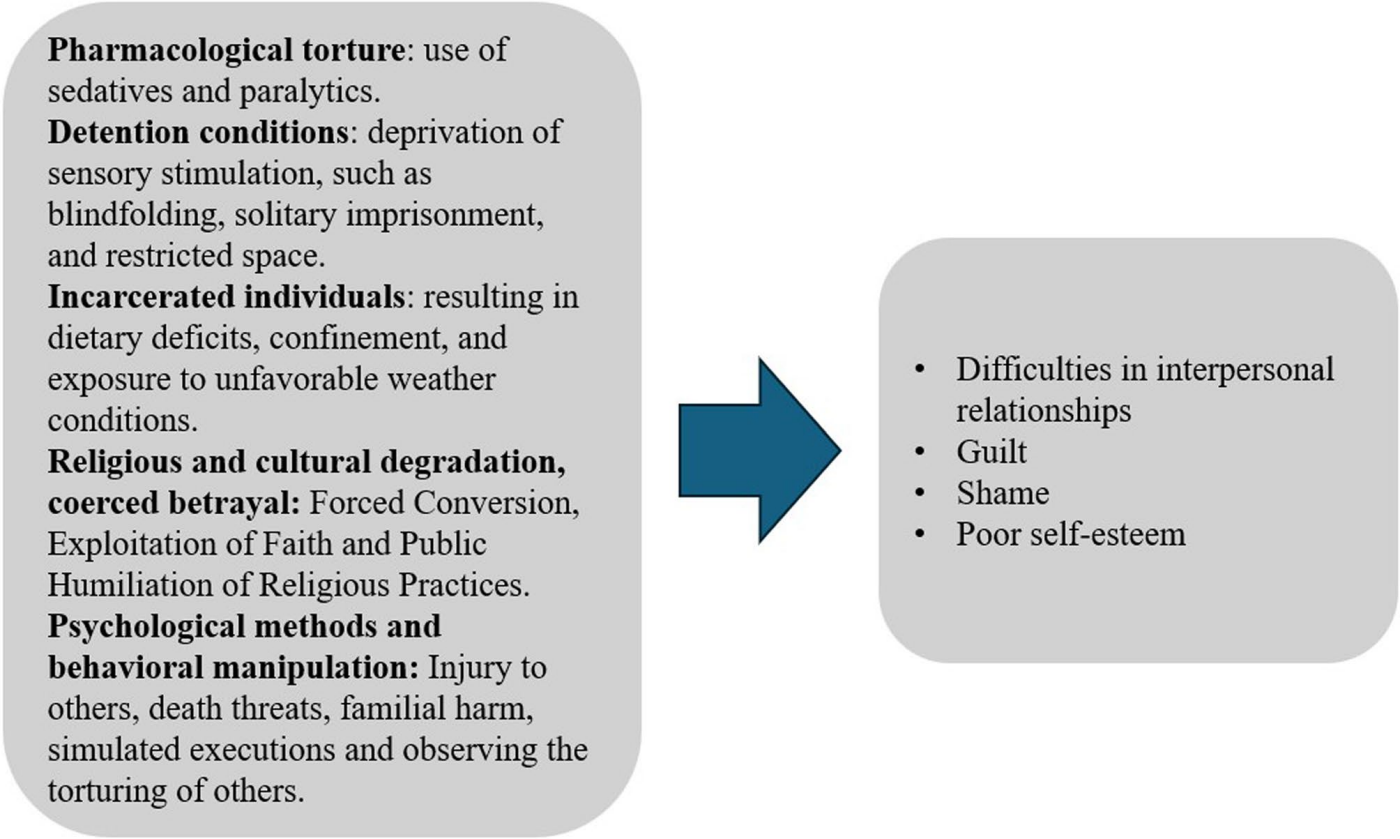
Torture methods that do not leave physical scars but have psychological consequences

Figure 1 illustrates several torture methods that, while not inflicting physical scars, result in psychological repercussions that disrupt interpersonal relationships (37) and cause feelings of guilt, (27, 38, 39) shame, (14, 27) and poor self-esteem (40) among survivors. These issues can lead to reluctance to share personal information, scepticism towards medical advice, and disengagement. Survivors may perceive health professionals as authority figures, triggering memories of their torturers. (17) Guilt can hinder open communication, leading to a lack of trust and a reluctance to seek help. Shame, a pervasive emotion, can lead to withdrawal and secrecy, obstructing comprehensive care. Poor self-esteem can also hinder assertiveness in interactions with the surgeon and other members of the surgical team.

The psychological consequences that the surgical team must consider as a background for the survivor’s behaviour in a clinical context include, but are not limited to, insomnia, nightmares, flashbacks, memory problems, lack of concentration, anxiety, and low mood [41]. Furthermore, there have been reports of elevated rates of substance abuse [42], psychosis [1], and suicidal ideation [43]. 

Although physical symptoms may have a psychological component, it is also essential to investigate any physical causes, such as those resulting from recurrent trauma or nutritional deficiencies [44, 45]. 

## The aftereffects of torture and the need for surgical treatment

Torture survivors often endure severe physical and psychological injuries [4, 27, 46] reflecting the brutality of the abuse [24, 47–52]. Long-term effects such as chronic pain and mobility impairments often require surgical intervention [18, 27, 28, 53–56]. Understanding these injuries is crucial for healthcare providers to meet survivors’ unique needs [30, 57–59], guiding both surgical and therapeutic strategies [33, 60]. 

Torture methods are deliberately designed to maximise suffering without causing death [61]. A systematic review of global torture prevalence and method frequency [4] found that perpetrators commonly use multiple techniques, thus resulting in complex and varied physical injuries. The consequences depend on the methods used and may involve structural damage, functional impairment, or both [4, 62]. It is often difficult to attribute specific injuries to specific acts due to the overlapping nature of torture practices, such as falanga (beatings to the feet), prolonged immobilisation, electric shocks, and sexual violence. Although most surgeons do not encounter recent torture cases due to delayed resettlement, they must be knowledgeable regarding torture-related symptoms to ensure accurate diagnosis and care [28]. 

The most common torture methods and their physical effects are summarised in Table 1 [4, 15, 24, 48, 52, 57, 63–73]. 

**Table 1:** Common torture methods and physical symptoms after torture

**Torture Methods**	**Acute Symptoms**	**Chronic Symptoms**
Beating	Bleeding, bruising, swelling, open wounds, fractures, dislocations, joint pain, pain, numbness, seizures, skin lesions	Scars, fractures, skeletal deformities, bone abnormalities, headache, back and joint pain, muscle pain.
Blunt Trauma	Bleeding, bruising, swelling, fractures, dislocations, and pain.	Scars, fractures, skeletal deformities, muscle pain and headache.
Forced Solar Gazing	Visual disturbances and burns to the retina.	Visual disturbances, retinal damage, and visual field loss.
Sexual Torture	Genitourinary system injuries (red or dark urine, dysuria, incontinence), vaginal discharge and bleeding, pain, sexual dysfunction.	Sexual dysfunction, chronic genital pain, erectile dysfunction, lower urinary tract symptoms, and gynaecological complaints.
Blow on the Ears	Tympanic membrane perforation, otitis, hearing loss, and auditory disturbances.	Auditive disturbances, chronic irritation, and conjunctivitis.
Telefono	Pain and auditive disturbances.	Auditive disturbances
Penetrating Injuries (gunshots, shrapnel, stab wounds, slash cuts)	Bleeding, open wounds, fractures, lacerations, and pain.	Scars, fractures, skeletal deformities, and chronic infection.
Asphyxiation (wet, dry, chemical)	Respiratory distress, impaired consciousness, seizures, burns, and nausea.	Chronic pulmonary complaints, fatigue, and gastrointestinal symptoms.
Suspension	Pain, muscle strain, fractures, joint pain, and skin lesions.	Contractures, muscle pain, skeletal deformities, and difficulty walking (altered gait, reduced stride, and walking speed).
Burns (chemical, thermal, cold, heat)	Burns (bulla or necrosis according to burn degree), Scar-related pain (due hypertrophic or keloid scars) skin lesions, and tissue damage.	Scars, chronic pain, muscle pain, chronic infections, and chronic skin irritation.
Submarino (submersion in contaminated water)	Nausea, vomiting, impaired consciousness, respiratory distress, and chemical injuries.	Chronic pulmonary complaints, gastrointestinal discomfort, chronic fatigue, and chronic infection.
Electric Shocks	Muscle spasms, burns, nerve damage, seizures, impaired consciousness, and pain.	Chronic fatigue, muscle pain, nerve damage, pain, and cardiovascular issues (palpitations, dyspnoea, and hypertension).
Forced Human Experimentation	Bleeding, fractures, skin lesions, burns, and pain.	Chronic infection, skeletal deformities, and gastrointestinal discomfort.
Traumatic Removal of Tissue/Appendages	Bleeding, shock, open wounds, pain, and fractures.	Scars, skeletal deformities, contractures, and chronic pain.
Extreme Physical Conditions (forced body positions)	Pain, fractures, joint dislocation, muscle strain, and fatigue.	Skeletal deformities, joint damage, and muscle pain.
Psychological Torture	Acute anxiety, panic attacks, confusion, and distress.	Long-term PTSD, depression, anxiety, chronic fatigue, insomnia and difficulty trusting others.
Direct Threats, Sensory Deprivation, Solitary Confinement, Mock Execution, Witnessing Torture, Uprooting	Anxiety, confusion, fear hallucinations, disorientation, and stress.	C-PTSD, anxiety, depression, emotional numbness, insomnia, and emotional instability.

The above table maps out the specific torture methods to Acute Symptoms (immediate physical and psychological effects) and Chronic Symptoms (long-term consequences), thus offering a more comprehensive understanding of the impacts of these forms of torture

## Identification of torture survivors in surgical settings

Identifying torture survivors in surgical settings is essential. It enables clinicians to recognise the complex physical and psychological effects of torture, provide TIC, and develop tailored surgical plans and pain management strategies. It also facilitates appropriate referrals to mental health and social support services [30]. Many torture survivors experience long-term psychological consequences such as post-traumatic stress disorder (PTSD), anxiety, and depression [1, 14, 74], along with chronic pain conditions [75]. These comorbidities can significantly affect both physiological and emotional responses to surgery, complicate postoperative recovery, and contribute to the recurrence of symptoms [28, 76]. For example, heightened threat sensitivity, hypervigilance, and altered pain perception, which are common in torture trauma-affected individuals [77] and other traumatised patients [78], may lead to increased procedural distress, difficulty with anaesthesia emergence, and prolonged recovery periods [28, 78]. 

Identifying a history of torture prior to surgery helps the surgical team manage coexisting conditions during the planned procedure. For example, anaesthesiologists can tailor anaesthesia plans to accommodate trauma-related sensitivities [78]. Awareness of a patient’s background (trauma history, cultural background, and prior healthcare experiences) also aids in anticipating and managing post-surgical complications, thus contributing to a safer recovery process [20]. The case study presented above illustrates this approach, where adjustments—such as clear communication, patient involvement, and environmental modifications—were made to ensure a more supportive experience [79]. 

Further, torture survivors may exhibit heightened mistrust towards surgeons and other medical personnel [12, 80]. Recognising their history and approaching them with sensitivity can help build trust and improve cooperation [12, 20, 81]. Although identifying torture survivors in surgical settings is essential, and despite the high likelihood of encountering such individuals among refugees and asylum seekers, healthcare professionals in surgical departments—including surgeons, anaesthesiologists, dentists, and nurses—often fail to do so before treatment begins [17, 27, 82]. Clinicians should be particularly vigilant with patients from persecuted minorities in regions where torture is common, particularly when signs such as chronic pain, multiple scars, or complex injuries are present [41, 74]. 

Torture survivors rarely volunteer their history [14, 19], often due to fear of disbelief or rejection [83], while clinicians may hesitate to ask due to uncertainty regarding how patients might react [20]. This mutual silence can hinder understanding of the patient’s condition. Proactively and sensitively asking about past physical or psychological assaults, including torture, while establishing trust, can offer relief to survivors and demonstrate genuine care and respect.

The voluntary disclosure of a torture story is also difficult for survivors due to the involvement of doctors in torture. In many instances, medical professionals may have been complicit in the acts of torture, either by providing medical treatment to prolong the torture or by participating directly in the abuse [84, 85]. 

The identification of torture survivors in a clinical context depends on proactive inquiry [86], which must be conducted sensitively and respectfully within a trauma-informed framework and healthcare providers need to be trained to recognise subtle behavioral and physical cues that may suggest a history of torture [32]. The use of trauma-informed screening tools can further support early identification in a non-invasive manner. Brief, self-administrated instruments, such as adapted versions of the Harvard Trauma Questionnaire [87] or the Refugee Health Screener 15 (RHS-15) [88] can be integrated into initial assessments, provided they are administered with care and informed consent. Equally important is the creation of safe opportunities for disclosure. This includes ensuring privacy, reducing time pressure, and clearly communicating the voluntary and confidential nature of any questions about past trauma [20]. Finally, appropriate communication is essential. The use of trained interpreters, attention to language barriers, and respect for the patient’s gender and cultural preferences all can contribute to building the trust necessary for disclosure and engagement in care [89]. 

## Physical examination prior to surgery

Conducting a physical examination in torture survivors prior to surgery requires a comprehensive, sensitive, and trauma-informed approach. Table 2 outlines key considerations across selected surgical specialties involved in the care of torture survivors.

**Table 2:** Considerations for physical examination in torture survivors

	**Considerations for Physical Examination in Torture Survivors**
Orthopaedics	Looking for signs of fractures, dislocations, and chronic pain conditions. Evaluating any old injuries or deformities that may indicate previous trauma. Paying special attention to areas commonly targeted during torture, such as the hands, feet, and joints. Assessing for chronic musculoskeletal pain and limited mobility, as these can be long-term consequences of physical abuse.
Gastroenterology	Assessing for abdominal pain, gastrointestinal bleeding, and signs of internal injuries. Being aware of symptoms that may result from stress or trauma, such as irritable bowel syndrome (IBS). Looking for signs of malnutrition or dehydration, which may be consequences of prolonged detention or poor living conditions. Considering psychological factors that can affect gastrointestinal health, such as stress-related ulcers or functional disorders.
Urology	Checking for urinary tract infections, incontinence, and any signs of trauma to the kidneys, bladder, or reproductive organs. Being mindful of any injuries related to sexual torture, such as genital trauma or sexually transmitted infections (STIs). Evaluating symptoms of chronic pelvic pain, haematuria (blood in urine), and any urinary retention issues, which could be indicative of past abuse or torture
Ear Specialist	Inspecting for hearing loss, damage to the inner and outer ear, and any signs of head trauma. Assessing for chronic ear infections or perforated eardrums that could result from beatings or loud noises. Being vigilant for balance issues or vertigo that could stem from inner ear damage. Looking for any facial injuries or scarring around the ears and head that may suggest previous violent encounters
Gynaecology	Conducting a sensitive and thorough pelvic exam, looking for signs of sexual assault or trauma. Assessing for infections, scars, and any other abnormalities that might result from torture. Ensuring a trauma-informed approach to avoid retraumatisation. Being mindful of psychological distress during the examination and providing a supportive environment. Screening for STIs, pelvic inflammatory disease (PID), and chronic pelvic pain, which may be long-term consequences of sexual violence. Considering discussing reproductive health concerns, including menstrual irregularities or fertility issues, that may arise from past trauma.|

## The role of surgical interventions in physical recovery

Surgical interventions for torture survivors encompass a range of procedures designed to address the physical injuries and trauma inflicted during torture [17, 28]. The surgical methods may vary significantly based on the severity and nature of the injuries sustained. In addition to orthopaedic interventions for fractures and joint dislocations, common surgical procedures include reconstructive surgeries that are designed to repair injury to soft tissues, bones, and nerves [24, 28, 48, 52, 65–67, 69]. Moreover, certain survivors may require surgeries to address internal injuries or organ damage that was caused by torture. In addition to restoring physical functionality, the objective of each surgical procedure is also to alleviate the psychological burden that is associated with the physical deformities and pain conditions. Surgical interventions play a crucial role in the rehabilitation of torture survivors by addressing physical injuries [28] and contributing to their overall recovery [57, 90]. Although there is limited literature on surgical care for torture survivors, some cases have been presented within a study. These cases report favourable outcomes, illustrating that surgical interventions can positively contribute to both the physical and psychological rehabilitation of survivors [71]. 

Certain physical sequelae of torture are unlikely to improve without surgical intervention, as they often involve significant structural or functional damage that cannot be adequately addressed through non-invasive treatments alone. One example of this is acid-related anal lesions described in a case study as a specific and severe consequence of torture in an individual survivor. In this instance, intentional exposure of the anal area to corrosive substances resulted in significant tissue damage, including burns, scarring, and strictures, with associated loss of normal function [91]. Without surgical correction, such injuries can result in debilitating conditions, such as faecal incontinence or severe difficulty in passing stools, which greatly impacts the survivor’s quality of life. Surgical repair can help restore normal function and relieve these complications [91]. Untreated physical injuries after torture can serve as constant reminders of the abuse, exacerbating PTSD and other mental health issues.

## The positive and negative outcomes of surgery in torture survivors

Surgery can have a range of outcomes for torture survivors, influenced by their unique social, physical, and psychological conditions. Understanding both the positive and negative potential outcomes can enable surgeons better prepare and support these patients through their surgical journey.

Table 3 presents some of the positive and negative Outcomes of Surgery in Torture Survivors.

**Table 3:** Positive and negative outcomes of surgery in torture survivors

**Outcomes**	**Details**
**Positive Outcomes**	
Alleviation of Pain	Successful surgical interventions can alleviate chronic pain from injuries sustained during torture, thus enhancing the survivor’s quality of life.
Physical Repair	Surgery can repair physical damage, restore mobility, and improve the ability to perform daily activities, thus leading to increased independence.
Psychological Benefits	Enhanced physical appearance and functionality can boost self-esteem and body image, thereby contributing to better mental health.
Empowerment	Successfully undergoing surgery can instil a sense of control and empowerment, aiding survivors regaining confidence in their ability to overcome challenges.
**Negative Outcomes**	
Post-Surgical Complications	Pre-existing injuries, scar tissue, and compromised health can lead to higher rates of complications and delayed healing processes.
Infections	Weakened immune systems and overall poor health increase the risk of infections, thus complicating recovery.
Retraumatisation	The surgical environment and procedures may trigger flashbacks or exacerbate PTSD symptoms, thereby causing considerable psychological distress.
Increased Anxiety and Depression	The inherent stress associated with surgery and recovery can heighten anxiety and depression, particularly in survivors with pre-existing mental health conditions.
Lack of Social Support	Lack of social support can delay recovery by making it more difficult for survivors to follow post-operative care and manage daily tasks during rehabilitation.
Financial Burden	The financial burden associated with surgery and recovery can be overwhelming for survivors with limited resources or access to healthcare.
Mistrust of Medical Professionals	Deep-seated mistrust of medical professionals can result in non-compliance with treatment plans and reluctance to seek follow-up care.
Cultural and Language Barriers	Cultural and language differences can lead to misunderstandings and hinder effective communication between the survivor and healthcare professionals.

### Positive outcomes

Successful surgical interventions have the potential to substantially alleviate chronic pain resulting from injuries sustained during torture, thereby significantly enhancing the patient’s quality of life. Such procedures can effectively repair physical damage, restore mobility, and improve the ability to perform daily activities, which in turn leads to increased independence for the survivor. Furthermore, the enhancement in physical appearance and functionality can have profound psychological benefits, such as boosting the survivor’s self-esteem and body image, ultimately contributing to better mental health. A successful surgery can also foster a sense of control and empowerment, thus aiding survivors in regaining confidence in their ability to overcome challenges.

### Negative outcomes

Despite these benefits, several negative outcomes must be considered. Torture survivors may present with a range of pre-existing physical and psychological health conditions—such as chronic pain, anxiety, depression, and sleep disturbances [2, 57]—that are known to influence postoperative recovery and pain outcomes [92]. Possible postoperative complications in torture survivors may not be directly caused by the experience of torture itself but rather mediated by the cumulative effects of associated health conditions—such as chronic pain, psychological distress, and poor general health. As existing literature reveals that poor baseline health can negatively impact surgical outcomes [92], this is particularly relevant for torture survivors, who often present with complex physical and psychological comorbidities [42, 93]. 

The risk of postoperative infections in torture survivors is another important negative outcome, which may be partially explained by immune dysregulation associated with PTSD. Research has revealed that PTSD can impair immune system function by disrupting stress hormone regulation and promoting a chronic inflammatory state [94]. This weakened immune response may compromise wound healing and increase susceptibility to infections in the postoperative period.

Further, insufficient social support constitutes another critical challenge, as it can delay recovery by making it difficult for survivors to follow post-operative care instructions, attend follow-up appointments, or manage daily tasks during rehabilitation. Without practical and emotional support, they may face challenges in medication adherence, wound care, mobility, and maintaining a healthy routine—all of which are critical for successful healing [17, 95]. 

A history of torture in an individual often leads to a deep-seated mistrust on others and also on medical professionals, which can result in non-compliance with treatment plans and reluctance to seek follow-up care [12, 17]. Cultural and language differences further exacerbate these issues, leading to misunderstandings and hindering effective communication between the survivor and the surgeon [19, 27, 96]. Moreover, it is also important for surgeons to distinguish torture from other types of interpersonal trauma when evaluating torture survivors. Torture is distinguished from other forms of interpersonal trauma by its distinctive characteristics, which include the intentionality, multiplicity, and extreme nature of the violations. These features frequently manifest themselves in the context of extended captivity [97]. Torture accounts frequently depict a sequence of highly unpredictable and adverse events, which include the deliberate infliction of pain and suffering, interspersed with rewards for cooperative behaviour [41]. This dynamic serves to destabilise reward systems and intensify anxiety [98]. The insidious nature of torture is not limited to physical suffering; it also encompasses the dissolution of social connections [99]. This is often a consequence of the participation of others in inflicting suffering, the use of psychological manipulation, or persistent social isolation [97, 100, 101] during torture. The models of torture effects emphasise disruptions in affective processes, particularly in areas related to social interaction and trust [13]. As a result, it is postulated that undergoing torture has a substantial impact on the brain systems associated with reward and anxiety in interpersonal relationships. This dynamic may interfere with the interaction between the survivor and the surgeon, and negative outcomes from surgery may be exacerbated by the survivor’s heightened fear and distrust [12, 13]. 

## The impact of chronic pain, PTSD, and amplified pain response on surgical treatment outcomes

Pain symptoms—including headaches, cervical and lumbar pain, bilateral shoulder pain, as well as discomfort in the musculoskeletal system and limbs—are commonly reported by many torture survivors. However, pelvic and urogenital pain may be underreported, potentially due to stigma, discomfort in discussing such issues, or a lack of awareness among healthcare professionals regarding the prevalence of these symptoms [102]. When addressing pain in torture survivors, it is essential to avoid presuming that the aetiology is solely psychological. A comprehensive assessment must be performed to ascertain the possible physical reasons for pain, as both physical and psychological elements may contribute to their distress [54, 56, 77]. 

Researchers have suggested that torture should be taken into account, as it frequently results in chronic pain in refugees [53]. Victims of torture may be unable to comprehend the source of their suffering or the methods by which it can be alleviated. Additionally, somatisation—in which pain symptoms mimic somatic disorders without an organic basis—is also prevalent [103]. Healthcare professionals, including surgeons, frequently encounter difficulty in comprehending this additional component [21]. In most cases, torture has usually occurred years before the patient’s pain manifestation, and central nervous system mechanisms are more likely to explain the pain [102]. Consequently, pain may confound the diagnosis of certain conditions that actually require surgical treatment in torture survivors [104]. For instance, chronic abdominal pain related to past beatings or stress positions may mask underlying adhesions or hernias. Similarly, persistent joint or back pain attributed to musculoskeletal trauma may delay the diagnosis of spinal injuries, disc herniation, or nerve compression syndromes [105]. In some cases, genitourinary pain resulting from torture may be misattributed to psychological distress, overlooking conditions such as urethral strictures or bladder injuries that require surgical intervention [71]. 

Torture survivors may have high rates of pain combined with PTSD, anxiety, and depressive symptoms [1]. Dysfunctional cognitions, such as negative trauma-beliefs and amplified pain response, may worsen the effects of depression and pain severity on PTSD [106, 107]. Moreover, changes in pain perception, chronic pain intensity, and traumatic suffering are interconnected. Due to alterations in pain perception, torture survivors have reported both hypersensitivity and hyposensitivity to pain. When exposed to powerful stimuli over their pain threshold, torture survivors have reported increased pain levels [17]. These individuals also had decreased pain inhibition and increased pain excitability where the nervous system becomes overly reactive to stimuli, further amplifying pain sensations [108]. The dysregulation in pain perception has direct implications for medical care, particularly during and after surgery. The reduced ability to inhibit pain and heightened sensitivity can lead to intensified pain experiences during procedures and recovery, thereby making pain management more challenging.

Understanding these links highlights the need for tailored pain management strategies for torture survivors [77, 108]. The approaches should address not only physical pain but also psychological and emotional suffering, recognising the complex interplay between trauma, mental health, and somatic symptoms in torture survivors [109]. Effective surgical interventions might include multimodal pain relief methods, TIC, and therapies targeting both physical and psychological aspects of pain.

Amplified pain response is another aspect that plays a significant role in predicting severe postoperative pain in torture survivors due to their unique psychological and physiological experiences with pain [106]. The combination of prior trauma, altered pain perception, and emotional distress creates a complex dynamic where amplified pain response exacerbates postoperative pain [110]. Postoperative pain from, for example, invasive dental operations, akin to other surgical interventions, may trigger horrific memories of torture [17, 111]. After surgery, surgical pain can trigger powerful reactions that may trigger torture memories [17]. 

Due the positive link between pre-operative pain severity and fear of surgery score [112], torture survivors who experience pain frequently may also be more anxious before surgery. This may also affect peri-and postoperative pain.

**Fig. 2:**
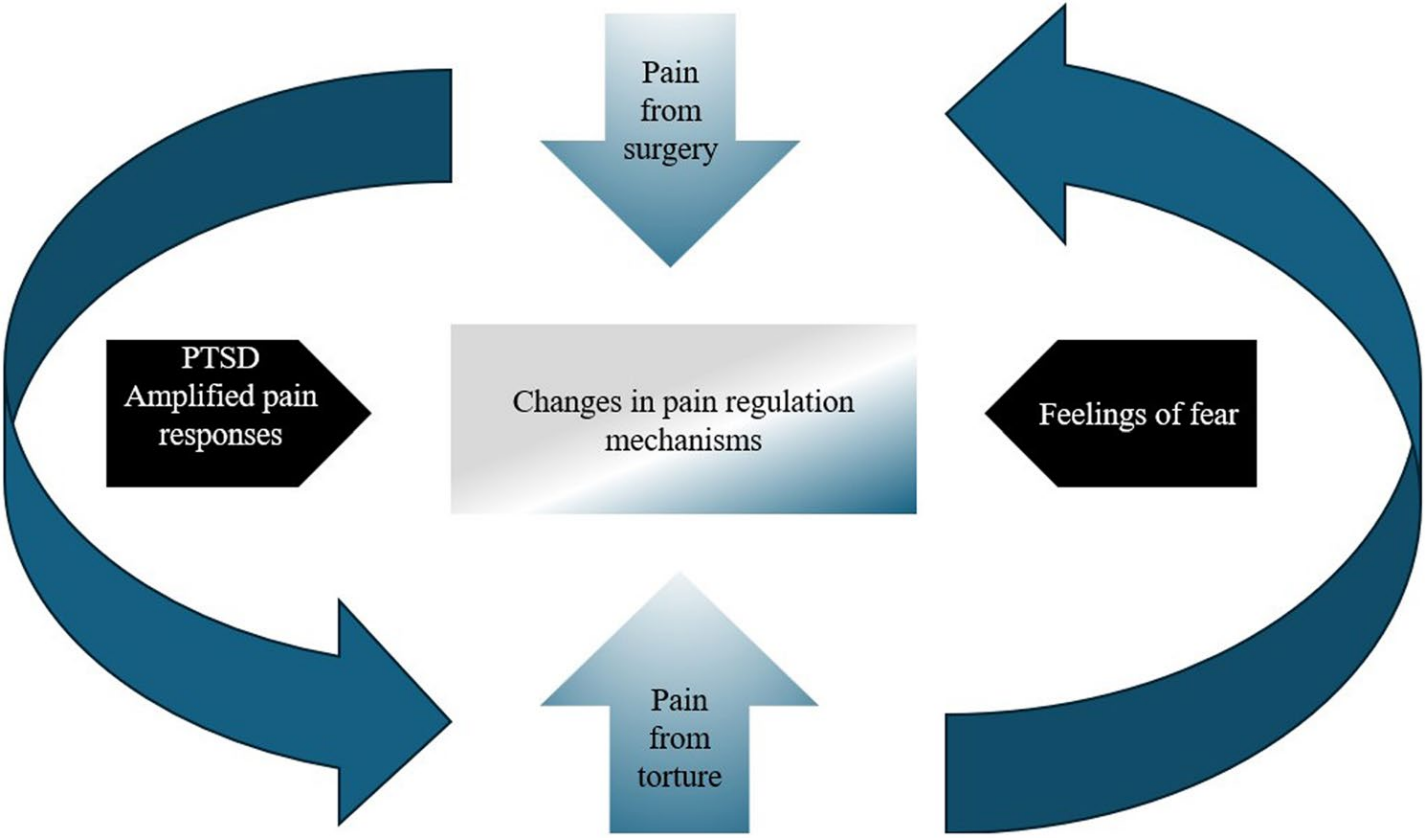
The complex dynamic between pain from surgery; pain from torture; and the role of fear, PTSD, and an amplified pain response

Further, post-traumatic stress disorder (PTSD) and torture-related suffering can also interact in complex ways when torture survivors undergo surgery [28]. This intricate dynamic often influences pain perception and may lead to misdiagnosis during surgical treatment [27, 28]. Survivors with PTSD, particularly those who have endured severe physical pain, such as torture, may interpret sensations and information regarding their bodies as inherently painful, akin to reliving the original traumatic event. The connection between torture and the personification of pain may be mediated by PTSD, thus shaping how survivors experience surgical pain [108, 113]. Somatic memory, the body’s ability to retain the imprint of traumatic experiences, can also play a significant role in these reactions [114]. As a result, postoperative pain can exacerbate retraumatisation by triggering vivid memories of torture and compounding the survivor’s physical and psychological distress [17]. This complex dynamic is depicted in Fig. 2.

The interplay between chronic pain, PTSD, and amplified pain response can significantly affect surgical outcomes due to the increased perception of pain post-surgery as well as higher levels of anxiety and stress. This may impede or delay healing, prolong recovery time, and potentially reduce the effectiveness of the surgical intervention [74, 106]. 

## Considerations for administering anaesthesia to torture survivors

There is limited literature that specifically addresses anaesthesia-related challenges in torture survivors. However, research on veterans, who, like torture survivors, may experience PTSD, provides valuable insights. These studies suggest that commonly used anaesthetic agents act on receptors in the amygdala and hippocampus to induce amnesia [115]. Upon discontinuation of anaesthesia, these areas of the brain become reactivated, which may lead to a heightened risk of emergence delirium (ED) in individuals with PTSD [115]. 

ED is characterised by an acute alteration in mental status during the immediate postoperative period. Clinical manifestations may include agitation, disorientation, distress, combativeness, restlessness, or confusion. Multiple factors contribute to ED, and patients with PTSD have been shown to experience it more frequently and with greater intensity than those without the diagnosis. This often results in prolonged stays in the post-anaesthesia care unit (PACU) and increased demands on healthcare staff and resources [115, 116]. Therefore, recognising PTSD in surgical patients is crucial. Patients with PTSD may also exhibit hyperarousal or dissociation during procedures, thereby complicating cooperation with staff and increasing the risk of procedural interruption or conversion to general anaesthesia [78]. 

Proactive identification, combined with tailored anaesthetic planning, can help reduce the incidence and severity of ED. This approach improves patient safety and psychological outcomes while also minimising postoperative complications, healthcare resource use, and risk to both patients and providers [78]. Given the high prevalence of PTSD among torture survivors [117], administering anaesthesia to this patient group requires careful consideration of their unique physical and psychological challenges. A few of the considerations that must be made are based in general literature on patients with PTSD and are presented in Table 4.

**Table 4:** Considerations for the administration of anaesthesia to torture survivors

**Category**	**Key Considerations**	**Details**
Pre-Anaesthetic Assessment	Medical History	Assessment of systemic conditions (e.g. airway injuries, cardiovascular or respiratory issues). [66] Pay close attention to respiratory function when caring for torture survivors, as prior episodes of asphyxiation—one of the most common and lethal forms of torture—may have lasting but subtle effects. Asphyxiation often leaves no external marks and may appear to resolve quickly, but it can result in undetected airway trauma, impaired respiratory function, or neurological sequelae due to cerebral hypoxia. Given the nonspecific physical findings, a detailed history and high index of suspicion are essential to ensure safe anaesthetic care. [118] Cardiovascular disease (CVD) has been associated with a history of water-based asphyxiation during torture. This association is particularly important for anaesthesiologists to consider, as underlying cardiovascular compromise may increase the risk of complications during the administration of anaesthesia. Thus, careful preoperative evaluation of cardiovascular function is essential in torture survivors with such histories, even in the absence of obvious clinical signs. [119]
	Psychological History	Conduct a thorough review of the medical record for evidence of PTSD, anxiety, and phobias. PTSD can significantly impact the patient’s response to surgical procedures, anaesthesia, and the overall perioperative experience. [14, 115]
	Physical Examination	Check for airway scarring, venous access challenges, and skin integrity. Examine for dental injuries, such as broken teeth or jaw fractures. Assess for oral infections, gum disease, and other dental conditions that could affect intubation and anaesthesia. [66]
	Imaging	Imaging for injuries (e.g. CT for airway concerns). Imaging may be essential in the evaluation of torture survivors, as not all physical sequelae are externally visible. Internal injuries—such as fractures, soft tissue damage, or organ trauma—may go undetected without appropriate radiological assessment, particularly when inflicted in ways designed to avoid visible marks. [66]
Psychological Factors	Trauma-Informed Approach	Explain procedures clearly; obtain consent; avoid triggering actions (e.g. restraints). [20, 33, 60]
	Sedation for Anxiety	Use mild sedatives (e.g. midazolam) for preoperative calming. [20] Midazolam may help reduce anxiety in torture survivors during procedures, [120] but its use requires caution. While it is effective in preventing strong reactions and fear during treatment, [121] it can worsen symptoms such as flashbacks in certain trauma-affected patients. This emphasises the importance of individualised sedation plans and TIC. [76]
Airway Management	Difficult Airway	Prepare for alternative techniques (fibreoptic intubation, tracheostomy) due to trauma history. Anaesthesiologists must exercise particular vigilance regarding airway management in torture survivors due to potential undetected or complex injuries. Dental torture—such as forced tooth extraction or electrical trauma—may result in fractured teeth, mucosal swelling, trismus, or temporomandibular joint dysfunction, all of which can complicate intubation. Additionally, prior strangulation may have caused laryngeal trauma, with possible delayed airway obstruction or vocal cord dysfunction. These injuries may not be externally visible, thus making a thorough history essential for safe anaesthesia planning. [66]
	Local anaesthesia	Explain the effects of local anaesthesia to avoid triggering panic during induction. It is important to explain the effects of local anaesthesia in advance, as the resulting numbness may resemble sensations experienced during torture, such as paralysis from hanging or restraint. [109, 122] Without preparation, this can trigger flashbacks or anxiety. Clear, reassuring communication helps reduce distress and supports a sense of control. [109, 111]
Pain Management	Multimodal Analgesia	Look for documentation on chronic pain management, which is common in torture survivors and may intersect with PTSD. Assess opioid tolerance or previous difficulties with pain control. Be aware of heightened pain sensitivity or exaggerated physiological responses (e.g. elevated heart rate, hyperventilation) due to PTSD. Combine NSAIDs, acetaminophen, and opioids. When discussing PTSD or trauma history with the patient, use compassionate, non-judgmental language to build trust. Explain how reviewing the record helps in creating a safer and more comfortable surgical experience. Share key details with the surgical and anaesthesia team to prepare for potential challenges, such as anxiety during induction or emergence from anaesthesia.
Postoperative Care	Calm Awakening	Create a peaceful environment to reduce distress during awakening.
	Psychological Support	Immediate access to mental health professionals if needed.
	Pain Control	Ensure effective pain relief with minimal side effects.
Multidisciplinary Care	Team Collaboration	Engage anaesthesiologists, psychologists, and surgeons for holistic care.
	Documentation	Ensure thorough medical and legal documentation, if required. Complete documentation for future care by updating the medical record with any new insights regarding PTSD symptoms or triggers identified during the preoperative process. Include notes on successful interventions (e.g. specific sedatives, support strategies) for future reference.

## The risk of retraumatisation during surgical care

Survivors of trauma, including torture, are at significant risk of being retraumatised during surgical care due to the reactivation of the original trauma during treatment. Retraumatisation occurs when medical procedures or environments inadvertently trigger memories of past trauma, thus leading to psychological and physiological distress.

Flashbacks, the most common manifestation of retraumatisation, involve intrusive memories that force the individual to relive traumatic events. These can be triggered by elements of the medical environment that resemble the torture experience, such as medical equipment, staff uniforms, prolonged waiting times, or the process of waking from anaesthesia. The causes of retraumatisation during surgical care have been extensively documented in the literature and reported by torture survivors undergoing surgical procedures [17]. A selection of these causes is outlined in Table 5.

**Table 5:** Causes of retraumatisation in surgical care

Environmental Triggers	
Clinical Settings	The sterile, impersonal nature of operating rooms can remind survivors of detention or interrogation rooms.
Sounds and Smells	Beeping monitors, the smell of disinfectants, or the sensation of a mask over the face may evoke past traumatic experiences.
Bright Lights and Darkness	Overhead surgical lights can remind survivors of sensory torture through light exposure. Darkness can remind of interrogation lighting.
Procedural Triggers	
Restraints	Being strapped to the operating table can replicate the experience of being physically restrained during torture.
Invasive medical procedures	Needles, intubation, or catheters may evoke feelings of violation or loss of control.
Pain or Physical Sensations	Pain during or after the procedure can act as a direct reminder of previous abuse.
Anaesthesia-Induced Vulnerability	The inability to move or speak under anaesthesia can be similar to the helplessness experienced during trauma.
Interpersonal Triggers	
Power Dynamics	The perceived authority of medical staff can echo the power imbalance during abuse.
Lack of Consent or Communication	Failing to thoroughly explain procedures can leave patients feeling out of control.
Rushed Interactions	Insufficient time for building trust can exacerbate fear and mistrust.
Psychological and Physical Impact of Retraumatisation.	
Psychological Reactions	Panic attacks or dissociation during or after care. - Heightened anxiety or resistance to medical interventions. - Exacerbation of PTSD symptoms, such as intrusive memories, flashbacks, or nightmares.
Physical Reactions	- Increased heart rate, blood pressure, or hyperventilation due to fear or stress. - Delayed recovery due to heightened stress response and poor cooperation. - Adverse reactions to anaesthesia or medications resulting from stress-induced physiological changes.
Strategies to Prevent Retraumatisation	
TIC	Recognise the potential for retraumatisation in all medical procedures. - Build trust with the patient through empathy, patience, and clear communication. - Validate the patient’s feelings and fears without judgment.
Preoperative Communication	- Explain every step: Clearly outline the procedure, purpose, and sensations the patient may experience. - Informed consent: Ensure the patient has the opportunity to ask questions and give voluntary consent. - Collaborative planning: Involve the patient in decision-making to give them a sense of control.
Environmental Modifications	- Allow the presence of a trusted companion or advocate in preoperative areas. - Minimise distressing noises and visual stimuli in the operating room. - Use calming techniques, such as dim lighting and quiet voices, during preparation.
Procedural Adjustments	- Avoid restraints: Use alternative methods (verbal de-escalation techniques, allowing patient choice and control) to ensure safety and stability without physical restraints unless absolutely necessary. - Preoperative Sedation: Administer mild anxiolytics to reduce anxiety and stress. - Gentle induction of anaesthesia: Use techniques to minimise fear during the induction phase, such as reassuring touch or distraction methods.
Anaesthesia Considerations	- Ensure the patient is fully unconscious before performing any invasive procedure. - Explain the effects of anaesthesia (e.g. muscle relaxation, temporary immobility) to prevent panic or confusion during emergence.
Postoperative Care	- Calm awakening: Ensure a quiet, supportive environment during emergence from anaesthesia. - Reorientation: Reassure the patient immediately upon waking by explaining where they are and what has occurred. - Early psychological support: Provide access to mental health professionals to address any distress or exacerbated PTSD symptoms.
Recognising Signs of Retraumatisation	a. During procedures - Resistance to medical interventions (e.g. pulling away, refusing care). - Panic or visible distress (e.g. hyperventilation, crying, or shaking). - Dissociation or unresponsiveness. b. Postoperative period - Increased agitation, confusion, or aggressive behaviour. - Recurrence of PTSD symptoms, such as nightmares or hypervigilance. - Avoidance of follow-up care due to negative experiences.

The risk of retraumatisation during surgical care is a serious concern for survivors of torture [17] and guidelines to prevent it have been developed by researchers [29]. By adopting TIC principles, customising surgical and anaesthetic approaches, and fostering clear communication, healthcare professionals can significantly reduce the likelihood of retraumatisation [32, 123]. 

## Communication and the use of interpreters

Effective communication is crucial when caring for torture survivors, as it lays a foundation of trust, accurate diagnosis, and appropriate care [124]. However, language barriers, cultural differences, and the psychological impact of trauma can complicate interactions in the clinical context [89]. In surgical services, the time that professionals spend with patients is limited across the preoperative, intraoperative, and postoperative areas [34]. Torture survivors often have complicated medical histories, yet professionals typically only focus on the aspects of history that are likely to influence the surgical procedure [20]. The surgeon and other members of the surgical team must make an active effort to understand the patient by accessing relevant aspects of their medical, psychological, and trauma history. This is essential to delivering person-centred care that responds to both physical and psychological needs [125]. 

Additionally, the absence of a common language can exacerbate stress and evoke feelings of loss of control among torture survivors. This mirrors the sense of powerlessness experienced during torture, potentially impacting their psychological well-being both before and after surgery [126]. More negative effects may occur if health professionals do not understand the patient due to language barriers. The usual practice is that interpreters are not used in the operating theatre, and interpreters are usually not present during the period of awakening from anaesthesia, which is the most sensitive period for the survivor with the risk of experiencing flashbacks [76]. A change in these routines may be necessary to provide adequate care to torture survivors and prevent complications such as retraumatisation during surgical care.

A few recommendations regarding the use of interpreters are presented in Table 6.

**Table 6:** Best practices regarding communication challenges when treating torture survivors

It is important to remember that the presence of an interpreter can also trigger strong reactions, particularly if the interpreter is from the same background as the torturer. It is important to be attentive of this dynamic, and it is important to ensure that the use of an interpreter is with the consent of the patient.	
**Communication challenges**	**Best practices**
Survivors may have difficulty articulating their symptoms due to language barriers, cultural nuances, or emotional distress. [17, 19, 89] **Challenges in Communication** - Language barriers: Many torture survivors are refugees or asylum seekers who may not speak the local language, thus making direct communication difficult. - Cultural nuances: Cultural differences in expressing pain, emotions, or consent can lead to misunderstandings. - Trauma-related factors: Survivors may struggle to discuss their experiences due to shame, fear, or difficulty revisiting traumatic events. - Fear of authority figures: Medical professionals can unintentionally remind survivors of past authority figures who inflicted harm, further inhibiting open communication.	Interpreters bridge the language and cultural gap, thereby enabling effective communication between the surgeon and other members of the surgical team and survivors. [127, 128] **Advantages of professional interpreters:** • Trained to handle medical and sensitive conversations with accuracy. • Maintain confidentiality and adhere to professional standards. **Best practices:** • Use interpreters with experience in TIC. • Avoid relying on family members, as this can compromise confidentiality and hinder honest communication. **Role of cultural mediators:** • In addition to language translation, cultural mediators help navigate cultural differences and ensure the survivor’s perspectives are understood. **Benefits of mediators:** • They provide context to expressions, gestures, or culturally specific behaviours that might be misinterpreted by healthcare professionals. **Respect for survivor autonomy:** • Allow survivors to express their preferences, including whether they feel comfortable with the interpreter provided. • Be sensitive to gender dynamics; certain survivors may prefer an interpreter of the same gender, particularly for sensitive topics. **Best practices for communication:** • Trauma-informed communication • Prioritise safety: Speak in a calm, non-threatening tone and avoid rushed interactions. • Seek consent: Always explain the purpose of questions or procedures and seek explicit consent before proceeding. • Empathy and validation: Acknowledge the survivor’s experiences without judgment, ensuring they feel heard and respected. • Use simple language: Avoid medical jargon, which may confuse or intimidate the patient. • Be patient: Allow time for survivors to process and respond, as they may need more time to articulate their thoughts or feelings.
Common Pitfalls to Avoid [128, 129]	
• Using untrained interpreters: Relying on untrained interpreters—such as family members or friends—can compromise trust, confidentiality, and the accuracy of information.	
• Ignoring non-verbal communication: Survivors may use non-verbal cues to express discomfort or emotions; these cues should not be overlooked.	
• Rushing the conversation: Pressurising survivors to disclose sensitive information can retraumatise them or lead to incomplete disclosures.	

Thus, it is evident that effective communication, supported by the appropriate use of interpreters, is vital when providing surgical care for torture survivors [124]. Professional interpreters and trauma-informed communication strategies can help overcome language and cultural barriers while fostering trust and safety among survivors [35, 130]. By creating a supportive and empathetic environment, surgeons, anaesthesiologists and the remainder of the surgical team can empower survivors to share their experiences of torture and participate actively in their care, ultimately improving the surgery’s outcomes and, thus, promoting healing [17, 20]. 

## Discussion

This study highlights the complex and often unpredictable challenges involved in providing medical care to survivors of torture, particularly when trauma histories are unknown or not fully disclosed at the time of treatment planning. Amin, a resettled refugee from Syria with a history of prolonged imprisonment and torture, experienced a significant psychological crisis during what was initially intended as a routine outpatient surgical procedure. His acute response emphasises the importance of TIC and the need for comprehensive preoperative assessments that take psychosocial history into account, particularly in patients from conflict-affected regions.

Amin’s acute agitation and expressions of feeling ‘trapped and exposed’ during the procedure likely represent a trauma reactivation in which aspects of the clinical environment (physical positioning, sensations, perceived loss of control) triggered an involuntary recall of torture experiences. This reaction was compounded by the initial lack of awareness among clinical staff regarding his background, thereby highlighting a critical gap in communication and trauma screening.

While the surgical procedure itself was technically uncomplicated, the psychological aftermath was profound. The use of propofol to manage his post-anaesthesia distress did not alleviate his symptoms and may have further disoriented him. The absence of an interpreter during his psychological crisis further contributed to his sense of isolation and may have intensified his confusion and vulnerability. Language barriers in such critical moments can severely undermine a patient’s sense of safety and trust, particularly in those already suffering from post-traumatic stress. The patient’s subsequent expressions of shame and his repeated apologies suggest a deep internalisation of trauma-related guilt or stigma, which are common in individuals with complex PTSD. Further, Amin’s refusal to engage with formal psychological services contrasted with his willingness to speak with a spiritual advisor; this emphasises the importance of culturally sensitive and flexible approaches to psychological support.

Overall, Amin’s case reinforces the necessity of systematic identification of survivors of torture within the healthcare system, the implementation of trauma-informed protocols across all levels of care, and the proactive use of interpretation services. It also highlights the importance of preparing staff to recognise and respond appropriately to signs of psychological distress in vulnerable patients, even during routine procedures.

For certain survivors of torture—particularly those who are religious or spiritual—faith-based or non-traditional interventions may play a more meaningful therapeutic role than conventional mental healthcare. The cultural significance of religion and spirituality, the support structures within religious institutions, and an understanding of the patient’s spiritual worldview are all crucial considerations when planning and delivering mental health services to refugee populations [131]. Recognising and integrating these dimensions into care can foster trust, reduce stigma, and enhance emotional healing in ways that align more closely with the individual’s values and coping mechanisms.

Further, the identification of torture survivors in surgical clinical settings is pivotal for providing tailored and effective care [20, 30]. Despite its importance, this process presents significant challenges, as members of the surgical team often hesitate to inquire about their patients’ trauma experiences [17]. Direct questions are not only essential for identifying survivors who might otherwise remain unnoticed but also provide an opportunity for these individuals to voice their needs and receive the appropriate care [132]. However, the timing, approach, and methodology of such questions remain a subject of debate [19]. Torture survivors themselves often express a desire for healthcare professionals to take the initiative in addressing their trauma history [17]. When healthcare professionals proactively ask about their patients’ trauma, they can provide immediate assistance and customise care to address the survivor’s specific needs. Yet, a major barrier to this practice is the lack of training among surgical teams, which leaves them ill equipped to ask sensitive questions or effectively handle disclosures [20]. This emphasises the necessity for comprehensive training programmes designed to educate healthcare professionals on how to approach these critical conversations in a sensitive and effective manner. Previous research has described various programmes designed to enhance the knowledge of healthcare professionals regarding the care of torture survivors. For example, one study highlighted a significant improvement in residents’ knowledge and self-efficacy related to the clinical evaluation of survivors following the implementation of a mandatory curriculum. This curriculum comprised a series of eight workshops, delivered in a single four-hour session [133]. Another study that provides guidance on preventing retraumatisation among torture survivors recommends that healthcare providers approach the topic of torture with both sensitivity and directness [20]. Clinicians are advised to ask about experiences of torture in a careful, respectful manner, avoiding euphemisms, while also signalling that they have the time and willingness to listen. This includes creating a calm, non-pressured environment in which the patient feels safe to disclose difficult experiences, if they choose to do so. Demonstrating patience and presence is essential to building trust and minimising the risk of retraumatisation during clinical encounters.

As the case presented in this article describes, the dual nature of trauma—encompassing both physical and psychological dimensions—demands an integrated, patient-centred model of care. Survivors of torture, such as Amin, may present with complex clinical profiles where physical symptoms are inseparably linked to psychological distress. Addressing only the physical aspects of care, without recognising the emotional and historical context, risks retraumatisation and poor outcomes. By fostering a safe, empathetic, and supportive clinical environment, surgical and medical teams are able to not only meet immediate health needs of survivors but also play a critical role in facilitating long-term psychological healing and recovery [35]. As described by Tremont (2021) in a study on TIC in surgical departments, primary care, psychiatry, and paediatrics have already begun to incorporate a broadened definition of trauma into patient care. However, to provide the maximum benefit to survivors of trauma generally and to torture survivors in particular, it is essential that the field of surgery also shifts towards a TIC approach [35, 60]. Surgical teams must recognise that the trauma associated with torture can interfere with interpersonal relationships and exacerbate the patient’s vulnerability. Although the primary responsibility of surgeons is to address physical injuries, an awareness of the psychological consequences of trauma is essential for providing comprehensive care [28, 35]. 

Surgical procedures inherently involve pain, which may trigger memories of past torture and heighten psychological distress in a survivor. Therefore, effective pain management is essential to minimise discomfort and prevent the reactivation of feelings and reactions related to the torture experiences [17]. In Amin’s case, the emergence of flashbacks, panic, and psychological distress following surgery illustrates how standard post-surgery management strategies may be insufficient for survivors of torture. Flashbacks can be triggered not only by psychological factors but also by physical stimuli, such as surgical pain, positioning, or the sensations associated with anaesthesia. This highlights the critical need for anaesthesiologists, surgeons, and the larger surgical team to recognise the altered pain perception common in individuals with PTSD. Research involving tortured ex-prisoners of war has revealed that PTSD can significantly mediate pain tolerance, with trauma impacting how the brain interprets and modulates bodily signals. For patients like Amin, this disrupted pain processing can contribute to heightened distress and re-experiencing of symptoms during or after medical procedures, thus reinforcing the need for trauma-informed, individualised pain management approaches [134]. To address these challenges, a comprehensive approach to pain management is essential. In addition to adapted pharmacological interventions, clear and empathetic communication regarding pain management plans and surgical procedures can provide reassurance, alleviate fear, and enhance the survivor’s sense of control, ultimately contributing to more effective and compassionate care.

Finally, it is essential that healthcare providers believe and validate the pain reported by torture survivors following surgery. Standard pain management protocols may be inadequate for this population due to the complex interplay between physical and psychological trauma. A trauma-informed, individualised approach to pain treatment is often necessary to address their unique needs effectively [20, 135]. 

The attitudes and behaviours of surgeons, anaesthesiologists, and other surgical team members may also be critical in shaping the experiences of torture survivors [17, 19]. Unfortunately, certain actions or dynamics, even if unintentional, can remind survivors of their torturers and lead to distress or mistrust [12, 80]. To address these challenges, healthcare professionals should adopt an approach characterised by empathy, trust-building, active listening, and education about the effects of torture. These principles can significantly improve interactions with survivors, leading to better health outcomes and more effective therapeutic relationships [35]. 

Research has been conducted on changing healthcare professionals’ attitudes toward traumatised patients, particularly through the implementation of TIC training programmes [136, 137]. These studies indicate that such training can enhance healthcare professionals’ understanding, empathy, and responsiveness to patients with trauma histories. A study evaluated the effects of a 1-day TIC training programme on the attitudes of 65 mental health professionals from 29 psychiatric hospitals [138]. Using a pre–post design with a three-month follow-up, the primary outcome was assessed with the Attitude Related Trauma-Informed Care Scale. Participants revealed a significant increase in favourable attitudes towards TIC. Additionally, 50% of participants reported implementing TIC practices in clinical settings by the follow-up. These findings highlight the effectiveness of brief TIC training in improving attitudes and encouraging practical application [138]. Another systematic review including 22 studies identified both barriers and facilitators to implementing such care, thereby highlighting the importance of training, organisational support, and leadership engagement in changing healthcare professionals’ attitudes and practices towards traumatised patients [139]. 

To implement TIC in surgical departments, existing guidelines regarding the treatment of torture survivors can serve as valuable tools. These guidelines can be supplemented or combined with digital solutions, such as electronic courses, to enhance accessibility and effectiveness. A study that presents guidelines to prevent retraumatisation of torture survivors during surgical care proposes this approach [20]. Combining existing guidelines with digital solutions can create a robust training programme that equips surgical teams with the necessary skills and knowledge to provide TIC. This approach ensures that all team members are well-prepared to understand and mitigate potential triggers and stressors during surgical care and create a clinical atmosphere that promotes healing and reduces anxiety for torture survivors [20]. 

## Contributions of the study

This study contributes to a deeper understanding of how surgical care can be improved for individuals with a history of torture. The findings highlight several key implications for healthcare professionals, particularly in surgical settings, thereby emphasising the need for trauma-informed, individualised, and ethically grounded approaches to care.

The described case highlights the therapeutic potential of culturally appropriate, non-traditional forms of psychological support, such as spiritual counselling, which may be more acceptable and accessible for certain patients. Therefore, healthcare systems must build capacity for flexible, interdisciplinary care pathways that respect the cultural, psychological, and existential dimensions of trauma recovery.

Overall, this case reinforces the ethical imperative for surgical teams to move beyond purely technical excellence and adopt a care model that prioritises the safety, dignity, and emotional well-being of torture survivors.

Enhancing surgical care for torture survivors requires the integration of trauma-informed principles into all stages of surgical care. The following implications highlight key areas where clinical practice can be adapted to better serve this vulnerable population:

1. Identification of torture survivors
  Early identification is essential, but many survivors may not voluntarily disclose their history due to fear, shame, or lack of trust. Clinicians should be trained to recognise indirect indicators—such as extreme anxiety, difficulty with physical touch or positioning, or avoidance of healthcare—and should create safe, non-judgmental opportunities for disclosure. Screening questions, when used, must be delivered with sensitivity and clarity, emphasising voluntary participation and confidentiality.
2. Trauma-Informed Communication and Trust-Building
  Surgical teams should allocate sufficient time for preoperative conversations to build a rapport and assess trauma-related vulnerabilities. Clear, compassionate communication that avoids medical jargon can help establish trust. Patients should never feel pressured to disclose traumatic histories, and staff should remain sensitive to signs of distress.
3. Enhanced Informed Consent Processes
  The informed consent process should go beyond procedural information to include a discussion of the patient’s sense of safety and autonomy. Offering the patient choices (e.g. in the sex of the care provider or positioning under surgery when possible) can help restore a sense of control that is often eroded by trauma.
4. Procedural Modifications to Reduce Retraumatisation
  Certain elements of surgical preparation and positioning may inadvertently trigger traumatic memories. Whenever possible, modifications should be made to avoid physical positions, lighting, or interactions that may resemble past torture methods. Maintaining privacy and minimising unnecessary exposure are critical to preserving patient dignity.
5. Anaesthesia Planning with Psychological Sensitivity
  Anaesthetic agents, particularly those associated with dissociative or disorienting effects (e.g. propofol), may exacerbate psychological distress in survivors of torture. Anaesthesiologists should be aware of these risks and consider patient preferences, psychological history, and possible reactions when selecting anaesthesia protocols.
6. Individualised Pain Management Strategies
  Standard postoperative pain protocols may be insufficient for torture survivors, who often have altered pain processing due to chronic consequences from trauma. Pain should be taken seriously and managed with multimodal approaches that incorporate pharmacological and non-pharmacological methods. Dismissal of pain reports can further erode trust and exacerbate distress.
7. Psychological Support in the Perioperative Period
  Psychological symptoms such as panic, flashbacks, or dissociation may occur during emergence from anaesthesia or while in recovery. Access to trauma-informed mental health support, spiritual care, or peer counselling should be available, and patients should be allowed to choose the form of support they prefer.
8. Postoperative Care and Follow-Up
  Recovery environments should be calm and quiet, minimising sensory overstimulation. Patients should be offered flexible and culturally sensitive follow-up options. Continuity of care with providers trained in trauma-informed approaches can promote long-term engagement and recovery.
9. Interdisciplinary Collaboration
  Multidisciplinary collaboration ensures that care is holistic, coordinated, and responsive to the patient’s cultural background, psychological needs, and lived experiences.

These adaptations are not only ethically and clinically warranted but are essential to providing equitable care that acknowledges the lasting impact of torture and the unique vulnerabilities of those who have endured it.

## Strengths and limitations

This narrative review addresses an underexplored area by synthesising diverse literature on surgical care for torture survivors. It offers practical, trauma-informed insights aligned with recent guidelines and identifies key gaps for future research. The use of an iterative search strategy contributed to the breadth and comprehensiveness of the review.

However, as a narrative review, the methodology does not meet the systematic rigor or reproducibility standards of systematic reviews or meta-analyses. The selection and interpretation of sources may be influenced by author bias, despite deliberate efforts to ensure relevance and diversity of perspectives. Further, the review is limited to published literature in English and indexed in major databases, which may have introduced language and publication bias and exclude grey literature or region-specific insights, particularly from low-resource settings.

Additionally, in certain themes, studies including case reports are cited to illustrate specific clinical challenges or consequences of torture. While these examples provide meaningful context, they inherently limit the generalisability of the findings. Due to the limited number of studies specifically focused on torture survivors, a few conclusions were drawn from research on generally traumatised individuals and patients with PTSD. This reliance highlights the pressing need for empirical studies and larger-scale research to develop a more robust, evidence-based foundation for surgical care practices tailored to torture survivors.

### Future perspectives and call for research

The future of surgical care for torture survivors should focus on systemic innovations and practices that enhance their psychological safety and well-being. Key priorities include developing and institutionalising trauma-informed protocols across preoperative, intraoperative, and postoperative stages, and customising care to address the specific needs of survivors, including strategies to prevent retraumatisation. Interdisciplinary collaboration, involving surgeons, anaesthesiologists, psychologists, and human rights organisations are essential for delivering comprehensive and empathetic care to torture survivors.

Despite progress in TIC, substantial gaps remain in understanding and optimising surgical care for this patient population. Future research should focus on evaluating the effectiveness of trauma-informed practices, preoperative counselling, and psychological interventions in improving surgical outcomes. Longitudinal studies are needed to explore the long-term social, physical, and psychological impacts of surgical care on torture survivors. Additionally, investigating the prevalence of re-traumatisation and its effects on surgical outcomes will be crucial in refining best practices.

## Data Availability

No datasets were generated or analysed during the current study.

## Abbreviations

CBC: Complete blood count
IBS: Irritable bowel syndrome
STIs: Sexually transmitted infections
PID: Pelvic inflammatory disease
PTSD: Post-traumatic stress disorder
TIC: Trauma-informed care
